# Controllable synthesis of layered black bismuth oxidechloride nanosheets and their applications in internal tumor ablation

**DOI:** 10.1093/rb/rbac036

**Published:** 2022-06-06

**Authors:** Qianlan Fang, Yu Xu, Lijia Luo, Chuang Liu, Zihou Li, Jie Lin, Tianxiang Chen, Aiguo Wu

**Affiliations:** Cixi Institute of Biomedical Engineering, International Cooperation Base of Biomedical Materials Technology and Application, Chinese Academy of Science (CAS) Key Laboratory of Magnetic Materials and Devices and Zhejiang Engineering Research Center for Biomedical Materials, Ningbo Institute of Materials Technology and Engineering, CAS, Ningbo 315201, P.R. China; University of Chinese Academy of Sciences, Beijing 100049, P.R. China; Cixi Institute of Biomedical Engineering, International Cooperation Base of Biomedical Materials Technology and Application, Chinese Academy of Science (CAS) Key Laboratory of Magnetic Materials and Devices and Zhejiang Engineering Research Center for Biomedical Materials, Ningbo Institute of Materials Technology and Engineering, CAS, Ningbo 315201, P.R. China; University of Chinese Academy of Sciences, Beijing 100049, P.R. China; Cixi Institute of Biomedical Engineering, International Cooperation Base of Biomedical Materials Technology and Application, Chinese Academy of Science (CAS) Key Laboratory of Magnetic Materials and Devices and Zhejiang Engineering Research Center for Biomedical Materials, Ningbo Institute of Materials Technology and Engineering, CAS, Ningbo 315201, P.R. China; University of Chinese Academy of Sciences, Beijing 100049, P.R. China; Cixi Institute of Biomedical Engineering, International Cooperation Base of Biomedical Materials Technology and Application, Chinese Academy of Science (CAS) Key Laboratory of Magnetic Materials and Devices and Zhejiang Engineering Research Center for Biomedical Materials, Ningbo Institute of Materials Technology and Engineering, CAS, Ningbo 315201, P.R. China; University of Chinese Academy of Sciences, Beijing 100049, P.R. China; Cixi Institute of Biomedical Engineering, International Cooperation Base of Biomedical Materials Technology and Application, Chinese Academy of Science (CAS) Key Laboratory of Magnetic Materials and Devices and Zhejiang Engineering Research Center for Biomedical Materials, Ningbo Institute of Materials Technology and Engineering, CAS, Ningbo 315201, P.R. China; University of Chinese Academy of Sciences, Beijing 100049, P.R. China; Cixi Institute of Biomedical Engineering, International Cooperation Base of Biomedical Materials Technology and Application, Chinese Academy of Science (CAS) Key Laboratory of Magnetic Materials and Devices and Zhejiang Engineering Research Center for Biomedical Materials, Ningbo Institute of Materials Technology and Engineering, CAS, Ningbo 315201, P.R. China; Advanced Energy Science and Technology Guangdong Laboratory, Huizhou 516000, P.R. China; Cixi Institute of Biomedical Engineering, International Cooperation Base of Biomedical Materials Technology and Application, Chinese Academy of Science (CAS) Key Laboratory of Magnetic Materials and Devices and Zhejiang Engineering Research Center for Biomedical Materials, Ningbo Institute of Materials Technology and Engineering, CAS, Ningbo 315201, P.R. China; Advanced Energy Science and Technology Guangdong Laboratory, Huizhou 516000, P.R. China; Cixi Institute of Biomedical Engineering, International Cooperation Base of Biomedical Materials Technology and Application, Chinese Academy of Science (CAS) Key Laboratory of Magnetic Materials and Devices and Zhejiang Engineering Research Center for Biomedical Materials, Ningbo Institute of Materials Technology and Engineering, CAS, Ningbo 315201, P.R. China; Advanced Energy Science and Technology Guangdong Laboratory, Huizhou 516000, P.R. China

**Keywords:** black bismuth oxidechloride nanosheet, photothemal therapy, tumor, photothermal conversion efficiency

## Abstract

The recently emerging bismuth oxyhalide (BiOX) nanomaterials are promising indirect band gap photosensitizer for ultraviolet (UV) light-triggered phototherapy due to their unique layered nanosheet structure. However, the low absorption and poor photothermal conversion efficiency have always impeded their further applications in cancer clinical therapy. Herein, BiOCl rich in oxygen vacancies has been reported to have full-spectrum absorption properties, making it possible to achieve photothermal property under near-infrared laser. Under 808 nm irradiation, the photothermal conversion efficiency of black BiOCl nanosheets (BBNs) is up to 40%. BBNs@PEG can effectively clear primary subcutaneous tumors and prevent recurrence, achieving good synergistic treatment effect. These results not only broke the limitation of UV on the BiOCl material and provided a good template for other semiconductor materials, but also represent a promising approach to fabricate BBN@PEG a novel, potent and multifunctional theranostic platform for precise photothermal therapy and prognostic evaluation.

## Graphical Abstract

**Figure:**
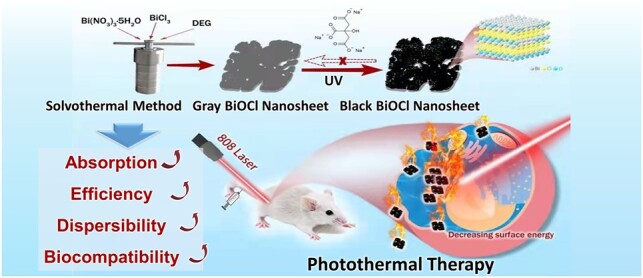

## Introduction

Near-infrared-light-(NIR, 700–1100 nm) mediated photothermal therapy (PTT) refers to the method that under the excitation of a certain wavelength light, photothermal conversion reagents absorb photons and release heat, thereby increasing the local temperature of the tumor, changing the environment of the tumor cells and making them degenerate, to achieve the purpose of treating the tumor [1, 2].

As a new type of tumor therapy similar to photodynamic therapy, PTT has attracted much more attention for its advantages of non-invasive, short treatment time, simple operation and remarkable curative effect [3, 4]. In addition, it can also be used as a supplementary therapy for cancer treatment such as chemotherapy and radiotherapy, which can improve the therapeutic effect by 50% [5, 6]. Photosensitizers play a decisive role in PTT and have become the focus of researchers all over the world.

The most widely studied photosensitizers are mainly divided into organic and inorganic photosensitizer [7–10]. Organic photosensitizer mainly refers to some NIR organic dyes [indocyanine green (ICG), IR825, etc.] and polymer nanomaterials (polyaniline, polypyrrole, dopamine, etc.) [8, 10]. The organic photosensitizer has good biocompatibility, but there are some obvious problems in their applications, such as low NIR absorption, relatively low photothermal conversion efficiency, easy photobleaching and poor material properties [11]. Inorganic materials are mainly composed of noble metal nanomaterials (gold nanorods, nanoshells, palladium, nanoparticles) [1, 2, 12–17], carbon nanomaterials (graphene oxide, carbon nanotubes, carbon quantum dots) [18–24], transition metal sulfides (copper sulfide, molybdenum sulfide, tungsten sulfide) [25–28], Prussian blue nanoparticles and black phosphate nanomaterials [29] and metallic oxide (bismuth oxide, titanium oxide) [30–33]. Compared with organic photosensitizer, inorganic photosensitizer has attracted more and more attention in the field of biomedicine due to their simple and controllable synthesis, high absorptivity, high photothermal conversion efficiency and good imaging function, and have broad application prospects in clinical trials later [7, 9, 34].

In recent years, as a new anti-tumor photosensitizer, inorganic bismuth-based materials have been extensively studied in the diagnosis and treatment of cancer due to their unique physical and chemical properties, strong NIR absorption capacity and excellent photothermal conversion performance [14, 35–40]. In addition, these materials have the advantages of simple synthesis method, low cost, long circulation time *in vivo* and good dispersion. Although Bismuth-based nanomaterials have been extensively studied in the field of catalysis, the research in biomedicine is still in its infancy. Although nanomaterials such as Bi_2_Se_3_ and Bi_2_S_3_ can obtain good photothermal effects in mouse tumor models, the cytotoxicity, instability and the poor photothermal conversion efficiency bismuth-based nanoparticles have been the main problems limiting their application in biomedical field.

BiOCl was originally one of the excellent materials in photocatalytic applications; there were a number of researchers using solvothermal reaction, photochemical reaction and high-temperature calcination reduction method, which endowed abundant BiOCl material defects, widening its absorption spectrum, from originally ultraviolet (UV) excitation to photocatalytic reaction under the visible light. OVs are the most prevalent and widely studied anion defects with a relatively lower formation energy on oxide surfaces. The OV-induced localized states could extend light response to visible/IR light and efficiently trap charge carriers, giving rise to the enhanced photoreactivity [38].

Our group first reported on the application of BiOCl-based nanomaterials in biomedicine [41]. The BiOCl nanoplates and BiOCl nanosheets (BBNs; 200 μg ml^−1^) achieved high PDT efficacies (about 90% tumor cell killing) under UV irradiation (low UV power density 2.2 mW cm^−2^, 30 min). Despite its high capability, the white BBN still got its shortage, such as the cytotoxicity, the instability and most important, the phototoxicity of UV light. Cheng *et al.* used the defect of BiOCl for tumor phototherapy, but a photothermal conversion efficiency of black BBNs was 13.9% in previous reported work, which was more lower than other inorganic nanomaterials for example gold nanoparticles [42]. It urgently to develop new synthesis of BBNs with high photothermal conversion efficiency for their biomedical applications.

**Figure 1.**
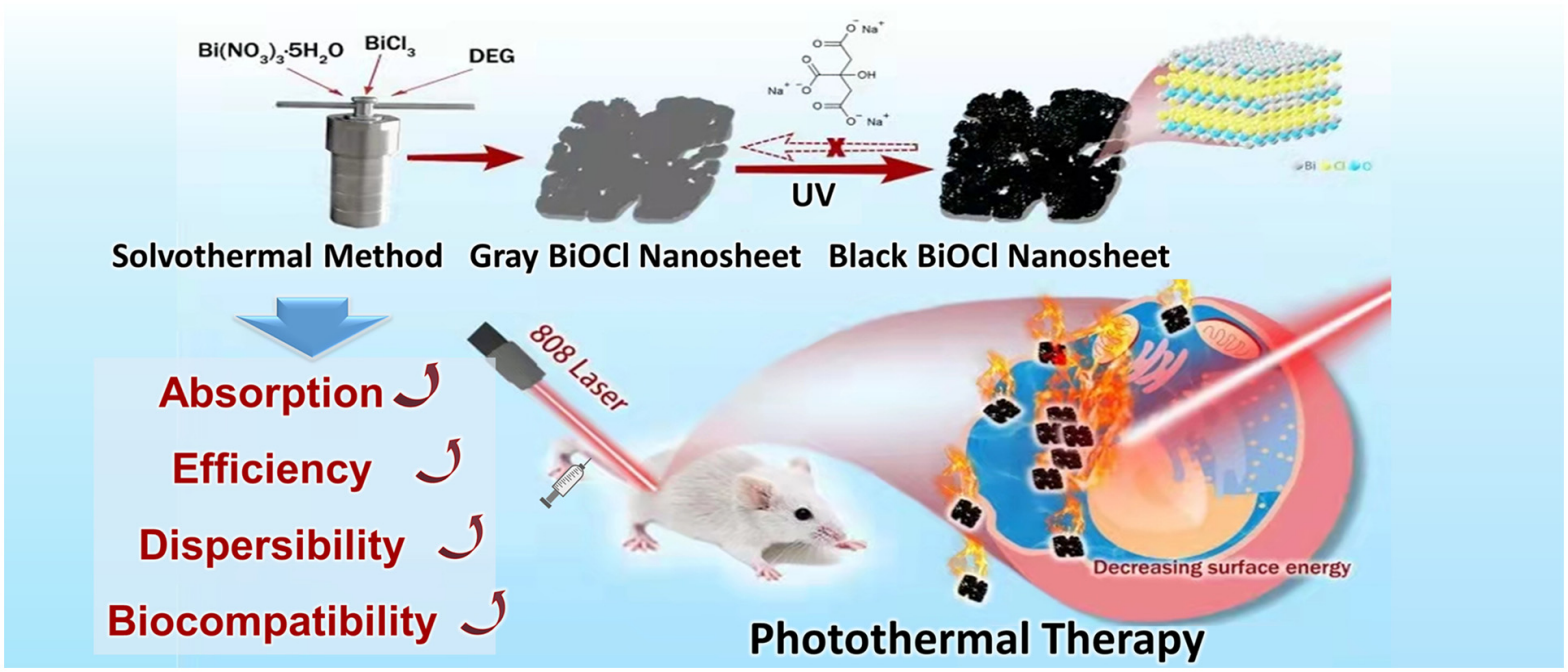
Schematic illustration of synchronized photothermal effect of BiOCl NSs with OV contents NIR (808 nm) irradiation.

In this study, we developed a solvothermal synthesis method for black BBN with sodium citrate modified and exposure to UV light (Fig. 1). We have found that the black BBN is not only with an excellent stability and inoxidizability but also an ideal photosensitizer with wider range of absorption, high conversion efficiency (up to 40%) and great biocompatibility. The improvement on the application of black BBN is critical in PTT of tumor, besides filling in gaps in photothermal application of wide-band gap semiconductor.

## Materials and methods

### Materials

The reagents used in this work have been listed in Table 1 and the instruments used in this work have been listed in Table 2 as following.

**Table 1.** Reagents used in this work

Regent	Purity	Source
Sodium citrate	99%	SIGMA
Tribasic dehydrate		
DEG	99%	Aladdin
BiCl_3_	AR	Aladdin
Chloral hydrate	AR	Aladdin
Bi(NO_3_)_3_·5H_2_O	99%	Aladdin
Mannitol	99%	Aladdin
Milli-Q water		
NaCl	99.5	J&K
Ethanolabsolute	AR	Sinopharm
PVP		Sinopharm
PEI	99%	Aladdin
FBS		Gibco
RPMI-1640		Gibco
MTT		
PBS		
KCl	AR	Sinopharm
Na_2_HPO_4_	99%	Aladdin
KH_2_PO_4_	AR	Sinopharm
DMSO	>99.8%	Aladdin
BSA		Gibco
Trypsin-EDTA		Gibco

**Table 2.** Instruments used in this work

Instruments	Model
NIR Laser Device	DS2-11312-403
Thermal Imager	JM500XC
Electronic Scales	ME104/02
Ultrasonic Cleaners	SB25-12DTDN
Magnetic Stirrers	HJ-6
Draught Drying Cabinet	DHG-9145A
Centrifuge	TGL-15B
Ultracentrifuge	ST 16R
Ultrasonic Processor	XC-CD
Carbon Dioxide Incubator	1501
Thermostat Water Bath	HH-6
Autoclave Sterilizer	MLS-3750
Refrigerator	BCD-226ST V
ELIASA	iMarkTM
Microscope	NIB-100
Fluorescent Microscope	3000B
Biosafety Cabinet	KS18
Ultra-low Tem Freezer	Forma 700
Low Speed Centrifuge	TDL-40C
TEM	JEM-2100F
ICP-OES	Spectro Arcosii
ZETA	Zetasizer Nano ZS
UV-Vis	T10CS
Photochemical Reactions	XPA

### Synthesis of black BiOCl intermediate

Black BiOCl intermediate was synthesized according to previously reported method with slight change. Briefly, 2 mmol (970 mg) Bi(NO_3_)_3_·5H_2_O was dissolved in 40 ml diethylene glycol in a 50-ml glass vial. When it was completely dissolved after ultrasonic treatment, 1 mmol (315.5 mg) BiCl_3_ was added in the vial. After ultrasonic treatment to transparent, the solution was transferred to 50 ml hydrothermal reactor, which was heated for 12 h under the temperature of 180°C degrees for hydrothermal reaction and then natural cooled down to room temperature naturally, the sediment was washed several times with water and ethanol and dried at 80°C for 10 h, BiO0.7Cl NSs was collected for the following experiment.

### Synthesis of black BBNs

Five milligrams intermediate mentioned above was dissolved into 10 ml aqueous solution of trisodium citrate with the concentration of 50 mg ml^−1^ (and now the concentration of intermediate is 500 μg ml^−1^). Then, the solution was separated to glass tubes and treated with the photochemical reaction instrument for 2 h, the UV light was provided by 500 W mercury lamp with UV filter. After that, the product was cleaned with water and ethanol for several times and collected by centrifugation and dried in oven at the temperature of 60°C.

BBN@PEG: 5 mg of BBN and 500 mg of PEG were added in 10 ml ethanol under magnetic stirring at ambient condition. The mixture was allowed to be incubated overnight for the PEG to decorate of the BBN. After that, the sediment was cleaned with water and ethanol for several times. When the reaction finished, sediment was gathered and purified by centrifugation at 10 000 rpm for 10 min with DI water and ethanol washing for several times. Then, synthesized BBN@PEG was re-dispersed in DI water.

### Synthesis BBN@PEG

One gram of PEG was completely dissolved in 20 ml absolute ethyl alcohol after 20 min ultrasonic processing. Then, 5 mg of as-prepared BBNs was dispersed in the solution. After stirring for 24 h, the product was cleaned with water and ethanol for several times and collected by centrifugation and resuspended in 10 ml of deionized water.

### Photothermal conversion experiment of BBN

Photothermal conversion efficiency was calculated by temperature elevation under the 808 nm laser irradiation. The above prepared BBN was dispersed into ultra-pure water with concentrations of 100, 200, 300, 400 and 500 μg ml^−1^ obtained through dilution. Take 1 ml solution of each concentration to transfer to the cuvette, using the wavelength of 808 nm infrared diode laser irradiation (this experiment using optical power density of 1 w cm^−2^) for 5 min. At the same time, adjust the light spot size to make it just cover liquid section and use the thermal imager real-time access to the temperature change of the sample solution during illumination. At the end of the experiment, the temperature and time curves of samples with different concentrations were obtained to calculate the photothermal conversion efficiency.

### Cell line and cell incubation

Murine breast cancer cell line 4T1 was cultured in RPMI 1640 medium (Gibco) supplemented with 1% penicillin/streptomycin and 10% heat-inactivated fetal bovine serum (FBS) in a 5% CO_2_ incubator at 37°C.

### *In vitro* cytotoxicity of BBN@PEG

One hundred microliters of 4T1 cells suspension was seeded into the 96-well plate per well at cell density about 1 × 10^4^ cells per well. *In vitro* cytotoxicity of BBN@PEG was determined by MTT assay. After overnight incubation, all the original medium were removed, and RPMI containing BBN@PEG nanosheet samples with different concentrations (0, 100, 200, 300, 400, and 500 μg ml^−1^) was added to each well, and after another incubation for 24 h. Subsequently, 10 μl of MTT (5 mg ml^−1^ in PBS) the above MTT solution was added to each well of the 96-well plate. After 4 h incubation, all the liquid in the 96-well plate was removed and 100 l of DMSO solvent was added to each well to dissolve the formazan crystals formed in cells. Finally, the absorbance was measured by a microplate reader at the wavelength of 550 nm, which is the characteristic absorption peak of formazan, to determine the relative number of remaining living cells in each well.

### *In vitro* PTT of BBN@PEG

The 4T1 cells were seeded in the 96-well plate with a density of 1 × 10^4^ cells per well for 24 h to make the cells stick to the wall. Then, all the original medium in the 96-well plate were removed, and RPMI-1640 containing BBN@PEG with different concentrations (0,100, 200, 300, 400 and 500 μg ml^−1^) was added to the corresponding wells according to the design and incubated for another 4 h. Each well was then washed with PBS and 100 μl fresh RPMI-1640 was added to each well. Then the cell plates of the photothermal treatment group were placed under 808 nm laser for laser irradiation. Experimental variables (laser power density and laser exposure time) were controlled. After the photo treatment, the cells were incubated for another 24 h. Then, 10 μl of the MTT solution dispersed in PBS at a concentration of 5 mg ml^−1^ was added to each well of the 96-well plate. Four hours later, all the liquid in the 96-well plate was removed and 100 μl of DMSO solvent was added to each hole to dissolve the formazan crystals formed in cells. Finally, using the microplate reader reads absorbance of each well at 550 nm, to determine the relative number of remaining living cells in each hole.

### Living-death cell staining experiment

Calcein-AM/PI double stain kit from Yeasen was used for live/dead cell staining. In a typical experiment, 4T1 cells were seeded in the 96-well plate with a density of 1 × 10^4^ cells per well for 24 h to make the cells stick to the wall. Then, all the original medium in the 96-well plate was replaced for RPMI containing BBN@PEG nanosheets with different concentrations (0, 100 and 500 μg ml^−1^) and incubated for another 4 h. Thereafter, the cells in each well were irradiated by 808 nm for another 5 min. After laser irradiation, the cells were cultured for another 24 h and stained by Calcein-AM/PI working solution for 15 min at 37°C. The fluorescence of cells was evaluated by fluorescent microscope.

### Animal models

Briefly, female BALB/c nude mice with body weight of 18–22 g aged 6 weeks were injected subcutaneously with 100 μl (1 × 10^6^) cells in RMPI were on the right flank of back, and all animal experiments followed were performed when the tumor reached about 100–150 mm^3^ [Tumor volume (V) = length ×width^2^/2]. All procedures used in this experiment were compliant with the local animal ethics committee.

### *In vivo* PTT of BBN@PEG

Tumor models were established on 5-week-old Balb/C females mice. Tumor size was measured with digital calipers. Tumor volume = tumor length*(tumor width^2^)/2. When the tumor diameter reached 3–4 mm, the mice were randomly divided into four groups, each group containing 6 mice. Mice were anesthetized by intraperitoneal injection of chloral hydrate solution with a mass fraction of 8%, and then intravenous (*i.v.*) injection of 50 μl sample aqueous solution (at a concentration of 2 mg ml^−1^) or 50 μl normal saline. The tumor site was exposed or not exposed to 808 nm laser (power density = 1 W cm^−2^) for 5 min. After the illumination, tumor size was measured by digital caliper for 14 days, and then the tumor volume was calculated by formula.

## Results and discussion

### Preparation and characterization of B-BiOCl nanomaterials

**Figure 2.**
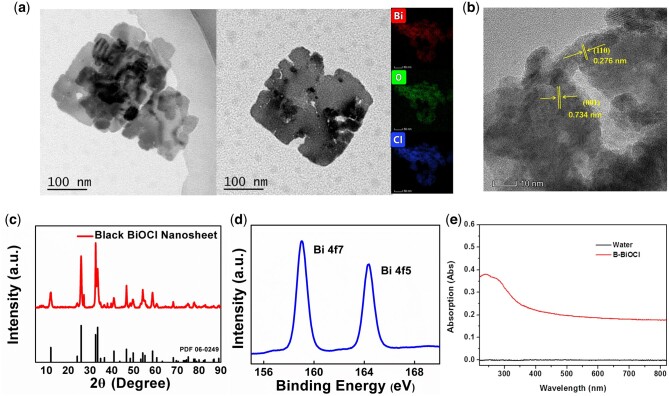
Synthesis and characteristic of BBNs. (**a**) TEM and EDS mapping of BiOCl. (**b**) HR-TEM images and of BBNs and crystal planes of 0.734 nm of (001), 0.276 nm of (110). (**c**) XRD patterns of BBNs. (**d**) XPS Bi 4f spectrum of BBNs. (**e**) UV-vis absorption spectra of BBNs.

As evidenced by the transmission electron microscopy (TEM) images (Fig. 2a), the BBNs had a uniform size (∼150 nm) with obvious surface defect. Then, we performed the high-angle annular dark-field scanning TEM (HAADF-STEM)-based elemental mapping (Fig. 2a) as well as energy-dispersive X-ray mapping, the images have further confirmed the structure and uniform distribution of element, which indicated BBNs crystal growth with high uniformity. High-resolution TEM (HR-TEM) image showed the lattice structure of BBNs with a lattice spacing of 0.734 nm (001) corresponding to the (001) crystal plane spacing of BBN, and 0.276 nm matched with the (110) crystal plane spacing (Fig. 2b). The X-ray powder diffraction (XRD) pattern showed the standard spectrum of the crystal BBN, which matched with the typical lattice planes (Fig. 2c). The peaks at 4f7 and 4f5 in X-ray photoelectron spectroscopy (XPS) are the characteristic peaks of bismuth element in BBN crystal (Fig. 2d), which further confirmed the successful fabrication of BBNs. In addition, the introduction of oxygen vacancy was further confirmed from the UV−vis−NIR absorbance spectra (Fig. 2e). Before the reduction reaction, the precursor of BBNs can only absorb UV light, and it is crucial to improve the bismuth-based.

### The photothermal conversion experiment of BBN

**Figure 3.**
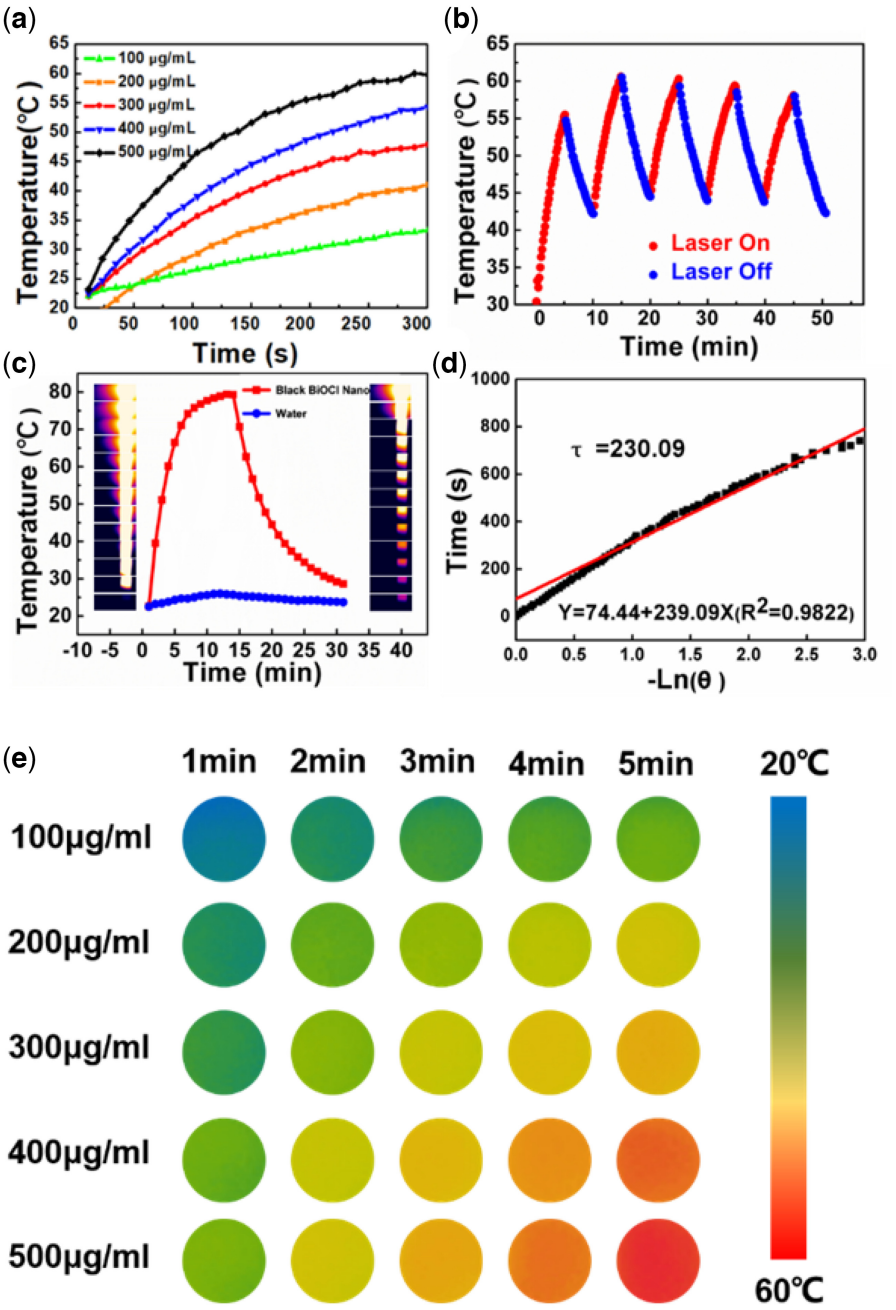
Photothermal effect and photothermal stability of BBNs. (**a**) Temperature elevation profiles of BBNs with 808 nm laser irradiation (1 W cm^−2^, 5 min). (**b**) The photothermal profiles of BBNs aqueous dispersions (200 μg ml^−1^) over five irradiation cycles. (**c**) The heating and cooling curves of BBNs (2 W cm^−2^). (**d**) The fitting curve of the absorption values at 808 nm of BBNs dispersions at different concentrations in water. (**e**) NIR photothermal images of BBNs aqueous solution under different irradiation conditions for 5 min under 808 nm laser irradiation (1 W cm^−2^, 5 min).

Photothermal conversion efficiency is an important criterion for evaluating the quality of photothermal conversion reagents. Encouraged by the high absorption in NIR region, the photothermal conversion properties of the materials were tested. The experimental results (Fig. 3a) showed that the aqueous solution of black BBNs heated rapidly under NIR excitation at 808 nm, with a concentration of 100–500 μg ml^−1^, when the laser power density was set to 1 W cm^−2^. The extinction coefficients of BBN@PEGs were determined to be 10.08 l g^−1^ cm^−1^ (Fig. 3d and e), indicating BBN@PEG’s high ability of photothermal conversion efficiency. The photothermal properties of BBN@PEG aqueous solution upon an 808 nm laser irradiation *in vitro* were systematically studied at different concentration under laser power density of 1 W cm^−2^ (Fig. 3d), evaluated by a digital NIR photothermal imaging system. The deionized water showed little change under laser irradiation; however, BBN@PEG presented a sharp contrast of temperature increasing to 70°C in 10 min. These results imply that the BBN@PEG has a stable ability to convert NIR energy to thermal energy rapidly and efficiently. Moreover, the photothermal conversion efficiency has been calculated to be ∼40% according to the reported methods (Fig. 3e). Then, the study on photostability of BBN@PEG was also clearly investigated that upon the continuous irradiation by NIR laser for 1 h. Under the laser illumination, BBN@PEG has no obvious impairment, indicating good photostability, which is very important toward further practical applications. Therefore, the excellent photothermal conversion efficiency and photostability of BBN@PEG make it to be a promising PTT agent with a remarkable potential.

### Biotoxicity test and PTT effect studying of BBN@PEG *in vitro*

**Figure 4.**
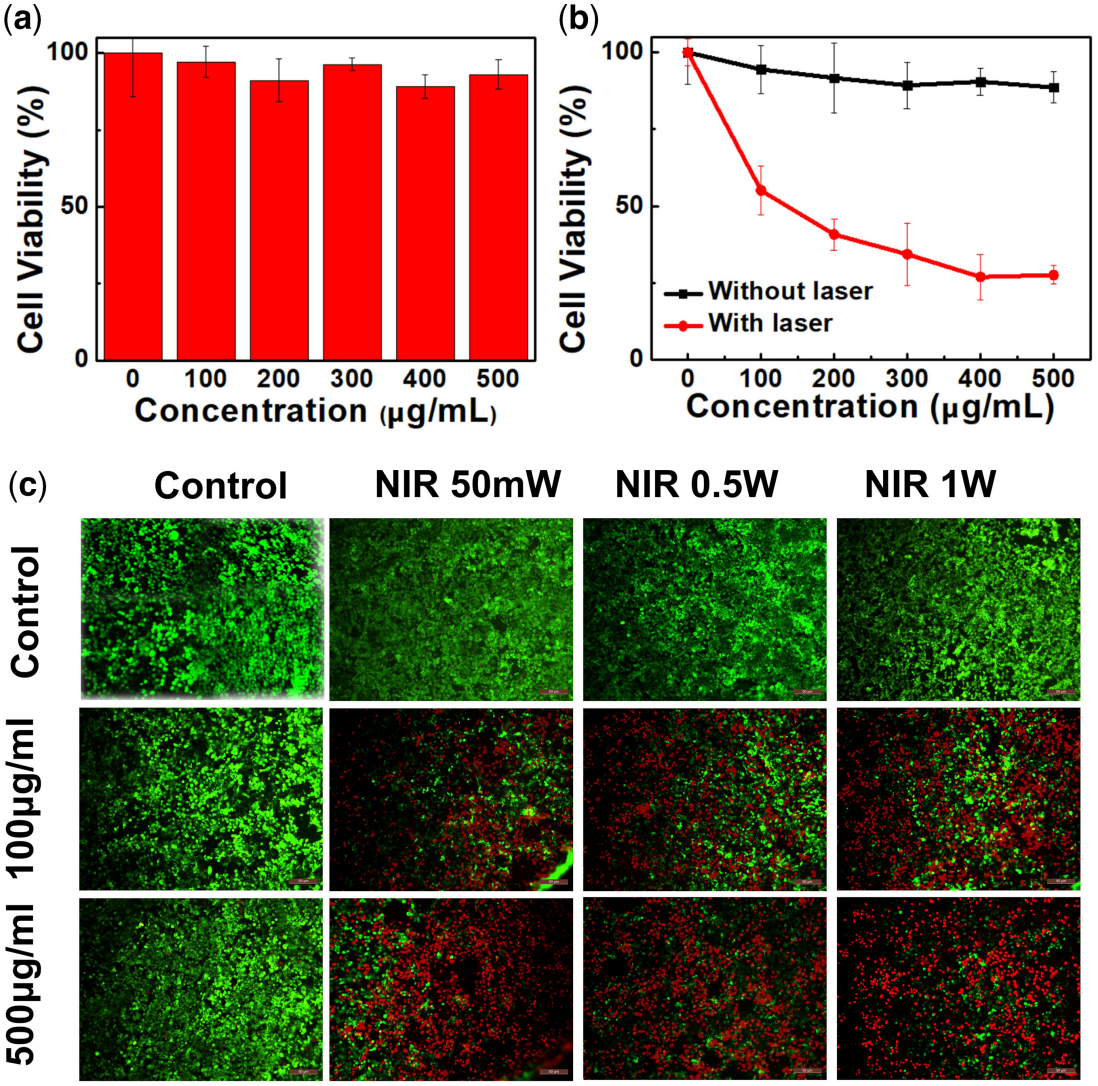
Biotoxicity test and PTT effect studying of BBN@PEG *in vitro*. (**a**) Cell viability assessments of 4T1 cells treated with BBN@PEG (0–500 μg ml^−1^) for 24 h without laser irradiation or (**b**) with 808 nm laser irradiation for 5 min (1 W cm^−2^). (**c**) Live/dead staining of 4T1 cells treated with BBN@PEG (100 and 500 μg ml^−1^) for 4 h with 808 nm laser irradiation for 5 min at 1 W cm^−2^.

Next, the cytotoxicity of these BBN@PEG is also a necessary part to study or further biomedical applications as well. The 4T1 cells that were incubated with BBN@PEG for different concentration, the standard 3-(4,5-dimethyl-2-thiazolyl)-2,5-diphenyl-2h-tetrazolium bromide (MTT) assay (Fig. 4c and d) and live/dead staining (Fig. 4e) were performed, indicating that BBN@PEG has a good biocompatibility. For example, even at a concentration as high as of 500 μg/ml, the cell viability of 4T1 cell remains more than 80% (Fig. 4c). Due to the high level of internalization property and nontoxicity, the *in vitro* PPT was carried on for forward verification of BBN@PEG therapeutic performance. The 4T1 cell was incubated with BBN@PEG for different concentration for 4 h, after washed off of BBN@PEG, the cells were exposed to 808 nm laser (power density = 1 W cm^−2^) for 5 min. Cells only exposed to laser remain alive, on the other side, the viability of cells incubated with BBN@PEG upon the NIR laser exposure has dramatically decreased to only ∼20% (Fig. 4d), meaning BBN@PEG high photothermal property on killing cancer cells upon NIR irradiation.

### Biotoxicity and test *in vitro*

**Figure 5.**
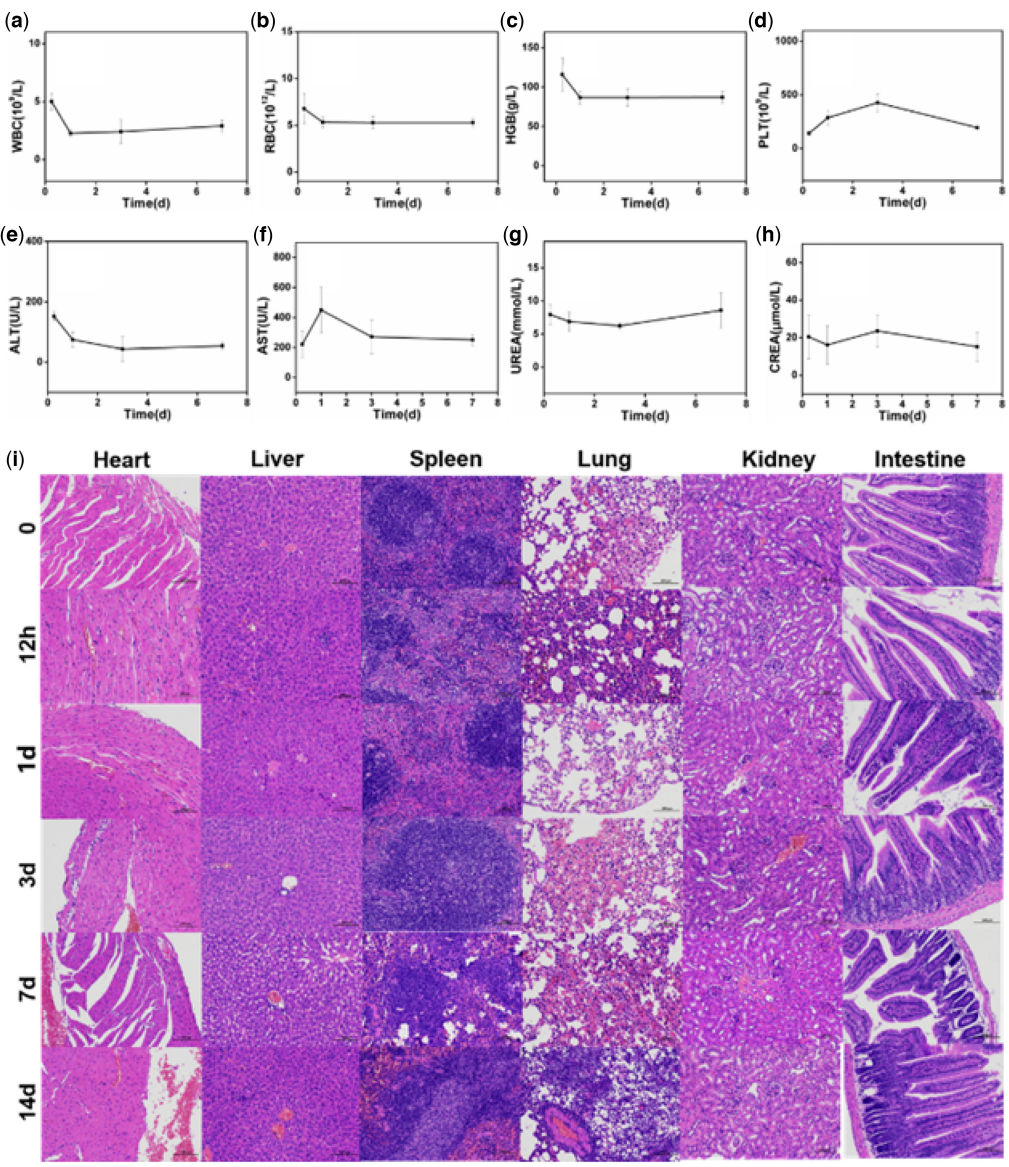
Hematological examination and pathological section of main organs of mice after *i.v.* injection of 50 μl BBN@PEG (2 mg ml^−1^). (**a–d**) Blood routine examination of blood cell (WBC), RBC, HGB and PLT. (**e–h**) blood biochemistry examination of AST, ALT, UREA and CREA.

*In vivo* cancer treatment experiment is an integral part for BBN@PEG to be nontoxic as well as biocompatible for organism. Blood routine examination (Fig. 5a–d) and blood biochemistry examination (Fig. 5e–h) were employed for nanomedicine safety monitoring. Healthy BALB/c mice were injected with BBN@PEG through caudal vein, and the blood sample was collected in next 6 h, 1, 3, 7 day for above-mentioned test. As showed in Fig. 5, the value of white blood cell (WBC), red blood cell (RBC), hemoglobin (HGB) and platelet (PLT) before and after injection almost unchanged, meanwhile, blood biochemistry examination is to test the levels of various ions, sugars, lipids, proteins, enzymes, hormones and metabolites present in the blood are measured. The main four parameters were chosen to monitor hepatorenal function. The value of aspartate aminotransferase (AST), alanine aminotransferase (ALT), blood urea (UREA) and creatinine (CREA) changed obviously 1 day after injection, nevertheless gradually return to normal in next few days, which notify BBN@PEG safety in hematology. To further testify if BBN@PEG were toxic to main organs *in vivo*, H&E stain was performed before and after injection in 6 h, 1, 3, 7, and 14 days. Comparing control group with injection group, there’s scarcely injury and inflammation was observed.

### PTT effect studying of BBN@PEG *in vivo*

**Figure 6.**
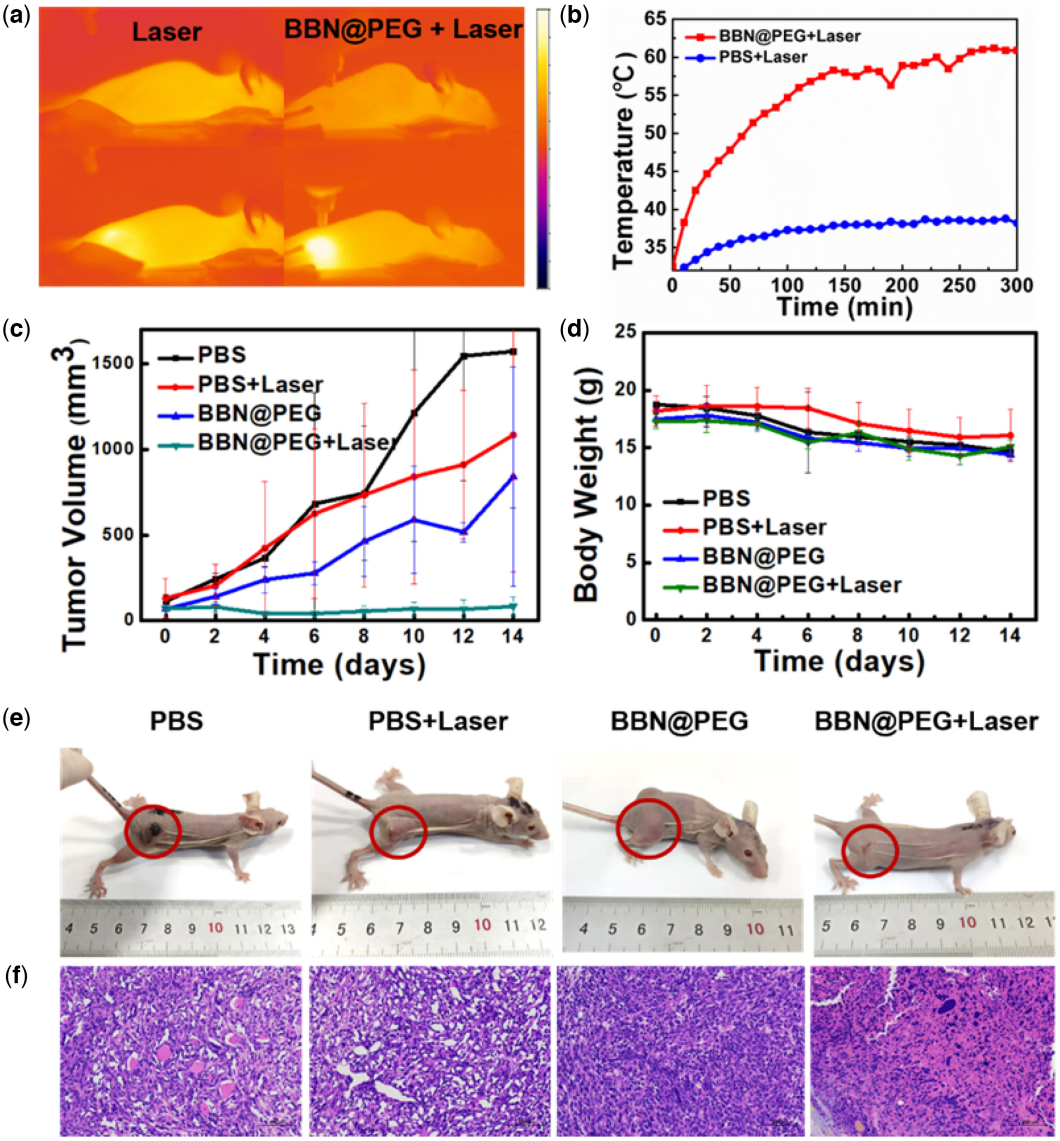
*In vivo* PTT effects of BBN@PEG in 4T1-tumor-bearing mice. (**a**) *In vivo* thermal imagery and (**b**) corresponding temperature variation curves of mice *i.v.* injected with PBS, or BBN@PEG NSs (2 mg ml^−1^) with 808 nm laser irradiation (1 W cm^−2^, 5 min). (**c**)Tumor volume curves and (**d**) body weight change curves of mice intravenously injected with PBS, or BBN@PEG NSs (2 mg ml^−1^) with or without 808 nm laser irradiation (1 W cm^−2^, 5 min) (**e**) the corresponding tumor photographs retrieved on the 16th day. (**f**) H&E staining sections of tumors of 4 groups dissected.

One of the best advantages of PTT is its much-limited damage to surrounding healthy tissue, controllable therapeutic process and avoiding the destruction induced by external laser radiation, which raise an essential precondition that the nanomedicine could gather to solid tumor based on the enhanced permeability and retention effect. Base on the above, the investigation of the BBN@PEG *in vivo* PTT was performed on 4T1 bearing mice model. To study the practicability BBN@PEG for oxygen-vacancy-enhanced PTT based on their outstanding performance of photothermal conversion, the 4T1 bearing mice were separated into following four groups until the tumor volume to ∼150 mm^3^, which are (i) PBS injection alone (PBS), (ii) laser irradiation alone (Laser), (iii) BBN@PEG *i.v.* injection alone (BBN@PEG) as well as (iv) BBN@PEG *i.v.* injection with laser irradiation (BBN@PEG + Laser). Figure 6a obviously shows that 4T1-bearing mice injected with PBS scarcely show response to the laser irradiation, which made a great contradiction to mice injected with BBN@PEG whose temperature raised from 30.9°C to 60.9°C in 300 s exposed to 808 nm laser (power density = 1 W cm^−2^). Next, 24 h after *i.v.* injection with or without laser irradiation (day 0), the tumor volumes (Fig. 6c) and the mice body weights (Fig. 6d) of mice were recorded every second day, which demonstrated the PBS, BBN@PEG and NIR laser irradiation all had neglectable effect on tumor growing. In BBN@PEG + Laser group, five in six mice had a full recovery without any evidence of recurrence. The relative survival rates (Fig. 6d) have been observed for 45 days, and there was still no death in BBN@PEG injection with laser irradiation group, further proving the distinct differences among all of the groups’ therapeutic efficiency. Figure 6f manifest representative pictures were taken of mice on the 14th day. H&E staining (Fig. 6g) was employed to verify the impairment and inflammation response on cellular level, presenting a large area of extensive necrosis of tumor tissue and necrotic nidus was eosinophilic, showing pink. Moreover, there was karyopyknotic in nucleus of necrotic tumor cells, the staining is deepened meanwhile fragmented and lysed, accompanied by a small area of bleeding and calcification after necrosis was also visible. Taken all together, BBN@PEG can accumulate in tumor region efficiently, presenting potent photothermal therapeutic effect, on which based NIR light-triggered PTT could be a reliable and efficient cancer treatment strategy.

## Conclusions

In summary, we synthesized a biocompatible, precise and multifunctional BBNs nanoplatform for PTT therapy of tumor, whose high photothermal conversion efficiency was enhanced by excellent oxygen vacancy defect structure. The as-prepared BBN@PEG process strong broad NIR absorbance, which is specifically devised for PTT and tumor ablation. The thermography for 4T1-bearing mice showed that tumor cell squint toward complete phagocytosis BBN@PEG, resulting in fantastic therapeutic effect for cancer cell. The results of 4T1 bearing mice with 100% survival rate show that the BBN@PEG had excellent diagnostic and therapeutic effects on precise PTT, which provided potential prospects for future clinical applications. Therefore, this work throws lights on the great potential of BBN@PEG-based nanomedicine for restraining solid tumor and encourages the further exploration of designing an oxygen-vacancy-enhanced PTT model for wide bandgap semiconductor for cancer therapy, which is believed to offer a promising clinical nanoplatform for efficient cancer therapeutics.

## Supplementary data

Supplementary data are available at *REGBIO* online.

## Supplementary Material

rbac036_Supplementary_Data
