# Supplementary material for: Controllable synthesis of layered black bismuth oxidechloride nanosheets and their applications in internal tumor ablation
Source: Regen Biomater. 2022 Jun 6;9:rbac036. doi: 10.1093/rb/rbac036 (PMC9348552; doi:10.1093/rb/rbac036)
Supplement: rbac036_Supplementary_Data [file rbac036_supplementary_data.docx]

Supporting Information

**Controllable synthesis of layered black bismuth oxidechloride nanosheets and their applications in internal tumor ablation**

Qianlan Fang,^a,c^ Yu Xu,^a,c^ Lijia Luo,^a,c^ Chuang Liu,^a,c^ Zihou Li,^a,c^ Jie Lin,^a,b^ Tianxiang Chen,*^a,b^ and Aiguo Wu*^a,b^

^a^ Cixi Institute of Biomedical Engineering, International Cooperation Base of Biomedical Materials Technology and Application, Chinese Academy of Science (CAS) Key Laboratory of Magnetic Materials and Devices and Zhejiang Engineering Research Center for Biomedical Materials, Ningbo Institute of Materials Technology and Engineering, CAS, Ningbo 315201, P.R. China.

^b^ Advanced Energy Science and Technology Guangdong Laboratory, Huizhou 516000, P.R. China

^c^ University of Chinese Academy of Sciences, Beijing, 100049, P.R. China.


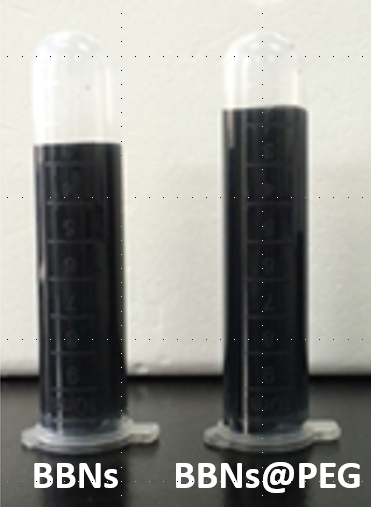


**Figure S1**. The water dispersion system of black BiOCl nanosheets (BBNs) was set for 24h.


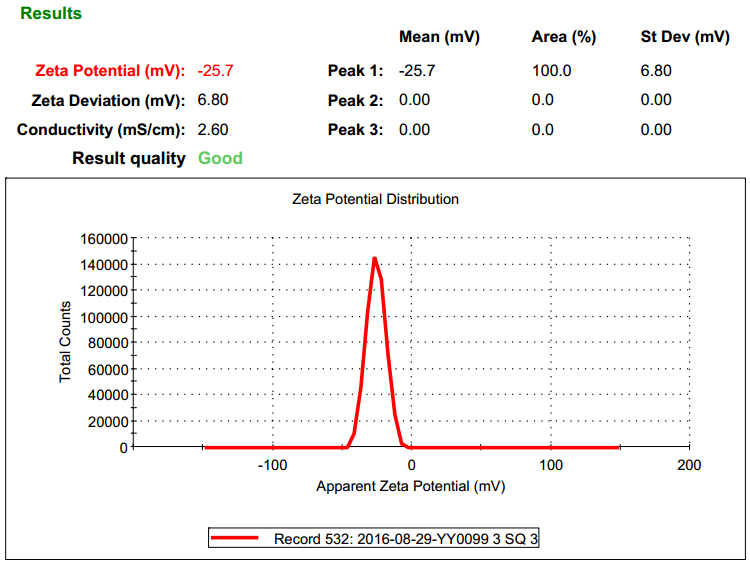


**Figure S2**. The Zeta potential test for BBNs.


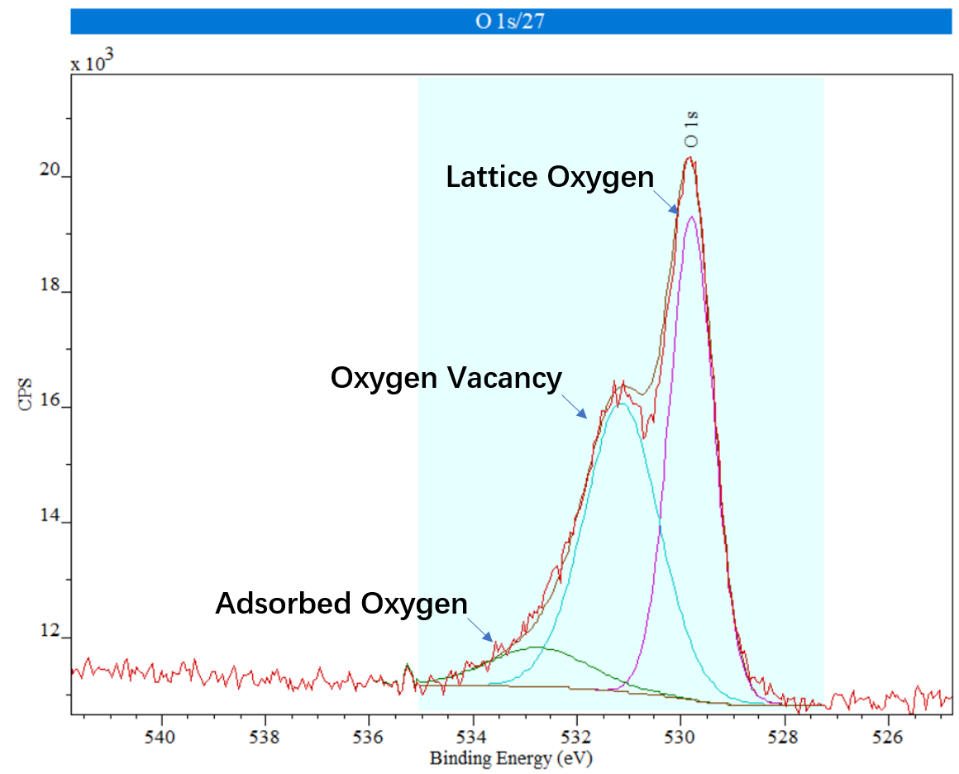


Figure S3. The XPS O 2s spectrum and the deconvoluted results of the BBNs.


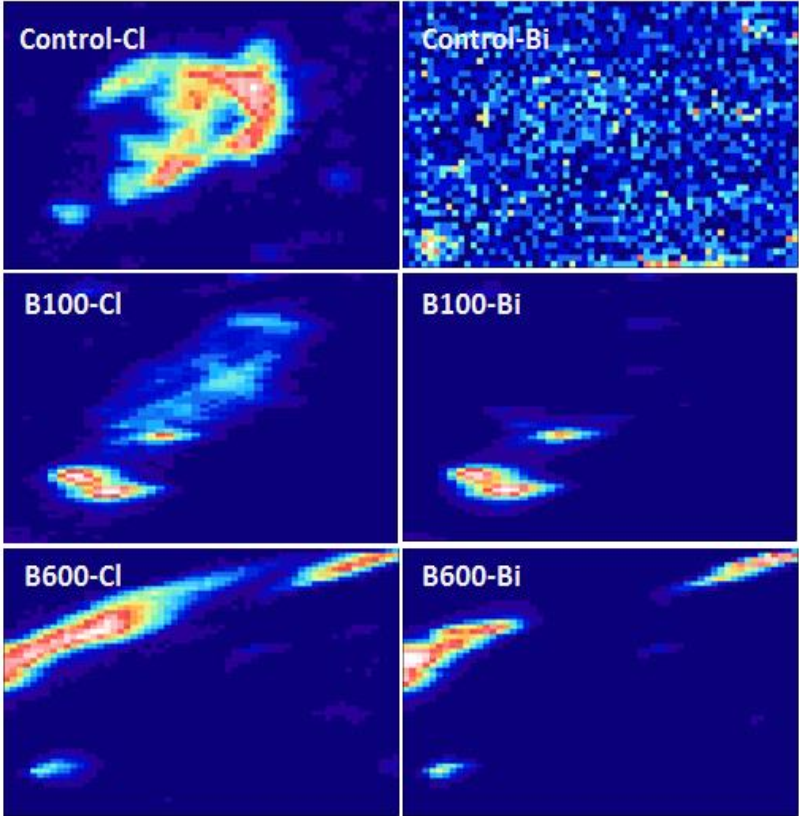


Figure S4. Cellular uptake ability of the BBNs.


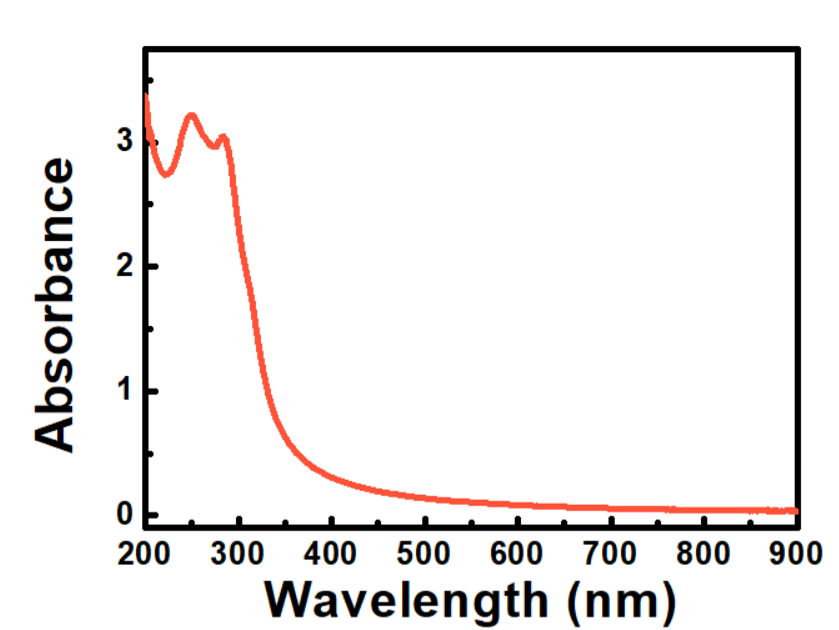


Figure S5. The UV-vis absorption spectra of BiOCl nanosheets.
